# Coupling of SK channels, L-type Ca^2+^ channels, and ryanodine receptors in cardiomyocytes

**DOI:** 10.1038/s41598-018-22843-3

**Published:** 2018-03-16

**Authors:** Xiao-Dong Zhang, Zana A. Coulibaly, Wei Chun Chen, Hannah A. Ledford, Jeong Han Lee, Padmini Sirish, Gu Dai, Zhong Jian, Frank Chuang, Ingrid Brust-Mascher, Ebenezer N. Yamoah, Ye Chen-Izu, Leighton T. Izu, Nipavan Chiamvimonvat

**Affiliations:** 1 Division of Cardiovascular Medicine, Department of Internal Medicine, School of Medicine, University of California, Davis, Davis, CA 95616 USA; 2 Department of Veterans Affairs, Northern California Health Care System, Mather, CA 95655 USA; 3 Department of Pharmacology, School of Medicine, University of California, Davis, Davis, CA 95616 USA; 4 Department of Physiology and Cell Biology, University of Nevada, Reno, Reno, NV 95616 USA; 5 Department of Biochemistry & Molecular Medicine, University of California, Davis, Davis, CA 95616 USA; 6 Health Sciences District Advanced Imaging Facility, University of California, Davis, Davis, CA 95616 USA

## Abstract

Small-conductance Ca^2+^-activated K^+^ (SK) channels regulate the excitability of cardiomyocytes by integrating intracellular Ca^2+^ and membrane potentials on a beat-to-beat basis. The inextricable interplay between activation of SK channels and Ca^2+^ dynamics suggests the pathology of one begets another. Yet, the exact mechanistic underpinning for the activation of cardiac SK channels remains unaddressed. Here, we investigated the intracellular Ca^2+^ microdomains necessary for SK channel activation. SK currents coupled with Ca^2+^ influx *via* L-type Ca^2+^ channels (LTCCs) continued to be elicited after application of caffeine, ryanodine or thapsigargin to deplete SR Ca^2+^ store, suggesting that LTCCs provide the immediate Ca^2+^ microdomain for the activation of SK channels in cardiomyocytes. Super-resolution imaging of SK2, Ca_v_1.2 Ca^2+^ channel, and ryanodine receptor 2 (RyR2) was performed to quantify the nearest neighbor distances (NND) and localized the three molecules within hundreds of nanometers. The distribution of NND between SK2 and RyR2 as well as SK2 and Ca_v_1.2 was bimodal, suggesting a spatial relationship between the channels. The activation mechanism revealed by our study paved the way for the understanding of the roles of SK channels on the feedback mechanism to regulate the activities of LTCCs and RyR2 to influence local and global Ca^2+^ signaling.

## Introduction

Small-conductance Ca^2+^-activated K^+^ (SK, K_Ca_2) channels are unique in that they are gated solely by changes in intracellular Ca^2+^ ^1,2^, hence, the channels provide a direct link between changes in intracellular Ca^2+^ and membrane potentials. Recent studies have provided strong evidence for the existence and functional significance of SK channels in the heart^3–7^. All three isoforms of SK channels (SK1, SK2 and SK3) are expressed in mouse and human cardiomyocytes^3,8^, and SK currents also exist in rabbit and rat ventricular myocytes^9,10^, canine pulmonary vein and left atrial myocytes^11^. Moreover, SK channels play significant roles in cardiac repolarization^3,8^, and are potential therapeutic targets against cardiac arrhythmias^4–6^.

Ca^2+^ signaling cascade represents one of the most important signaling pathways that controls excitability, excitation-contraction coupling, and contractility of cardiomyocytes, as well as regulates mitochondrial function, cell death, and gene transcription^12^. Intracellular Ca^2+^ is tightly controlled on a beat-to-beat basis by multiple molecular complexes to mediate micro- and nano-domain Ca^2+^ concentrations critical for the precise regulation of diverse function of Ca^2+^. Main ion channels and transporters of Ca^2+^ into and out of the cells and intracellular Ca^2+^ stores include Ca^2+^ channels, Na^+^/Ca^2+^ exchanger, ryanodine receptor 2 (RyR2), and sarcoplasmic reticulum (SR) Ca^2+^-ATPase. The function of these molecules are orchestrated by a network of subcellular signaling molecules including calmodulin (CaM), Ca^2+^/CaM-dependent protein kinase II (CaMKII), phospholamban (PLB), cAMP, and protein kinase A (PKA).

Similar to Ca^2+^ channels and transporters, gating of SK channels may be regulated by a network of proteins that participate in intracellular Ca^2+^ regulation. Among them, Ca^2+^ channels and RyR2 represent the two key molecules that may regulate SK channel gating because of their spatial proximity to SK channels in cardiomyocytes. Our previous studies revealed that cardiac SK2 channels coupled with L-type Ca^2+^ channels (LTCCs) through a physical bridge, α-actinin2, suggesting that LTCCs may functionally regulate SK2 channels by providing local Ca^2+^ domain to activate the SK channels^13^. However, a recent study suggests that RyR2-mediated SR Ca^2+^ release is both necessary and sufficient for SK channel activation, using SK2-overexpressed rat ventricular myocytes^14^. Another study reported that inhibition or knockdown of RyR2 or depletion of SR Ca^2+^ store significantly reduced SK currents in mouse atrial myocytes^15^. Both studies support the importance of RyR2 in the activation of cardiac SK channels.

In addition, previous studies have suggested the dichotomy in the regulation of SK compared to the large conductance Ca^2+^-activated K^+^ (BK) channels in neuron. BK and SK channels are regulated by nano- *vs*. micro-domain intracellular Ca^2+^, respectively^16^. However, the precise mechanism for the activation of cardiac SK channels in cardiac myocytes remains incompletely understood. The lack of spatial localization of SK channels, Ca^2+^ channels, and RyR2 in cardiomyocytes further hinders the understanding of SK gating mechanisms in cardiomyocytes. Indeed, dysregulation of Ca^2+^ channels and RyR2 can result in life-threatening cardiac arrhythmias, both in hereditary cardiac arrhythmia syndrome or acquired heart diseases. New insights into the mechanistic links underlying the exquisite regulation of SK channels are critically important not only in normal but also in diseased hearts.

In this study, we used stimulated emission depletion (STED) microscopy to acquire high resolution images to precisely localize SK channels in relation to Ca^2+^ channels and RyR2 in cardiomyocytes. We functionally test the source of local Ca^2+^ domain required for the activation of SK channels in rabbit ventricular myocytes. Our data provide high resolution quantification of a protein complex including SK2, LTCC, and RyR2 in cardiomyocytes. The distribution of nearest neighbor distances between SK2 and RyR2, as well as SK2 and LTCC, is bimodal, suggesting a spatial relationship between the channels. Our study reveals that Ca^2+^ influx through LTCCs and Ca^2+^ release *via* RyR2 provides the immediate and efficient Ca^2+^ microdomain for the activation of SK channels.

## Results

### Apamin-sensitive SK currents in rabbit ventricular myocytes

**Figure 1:**
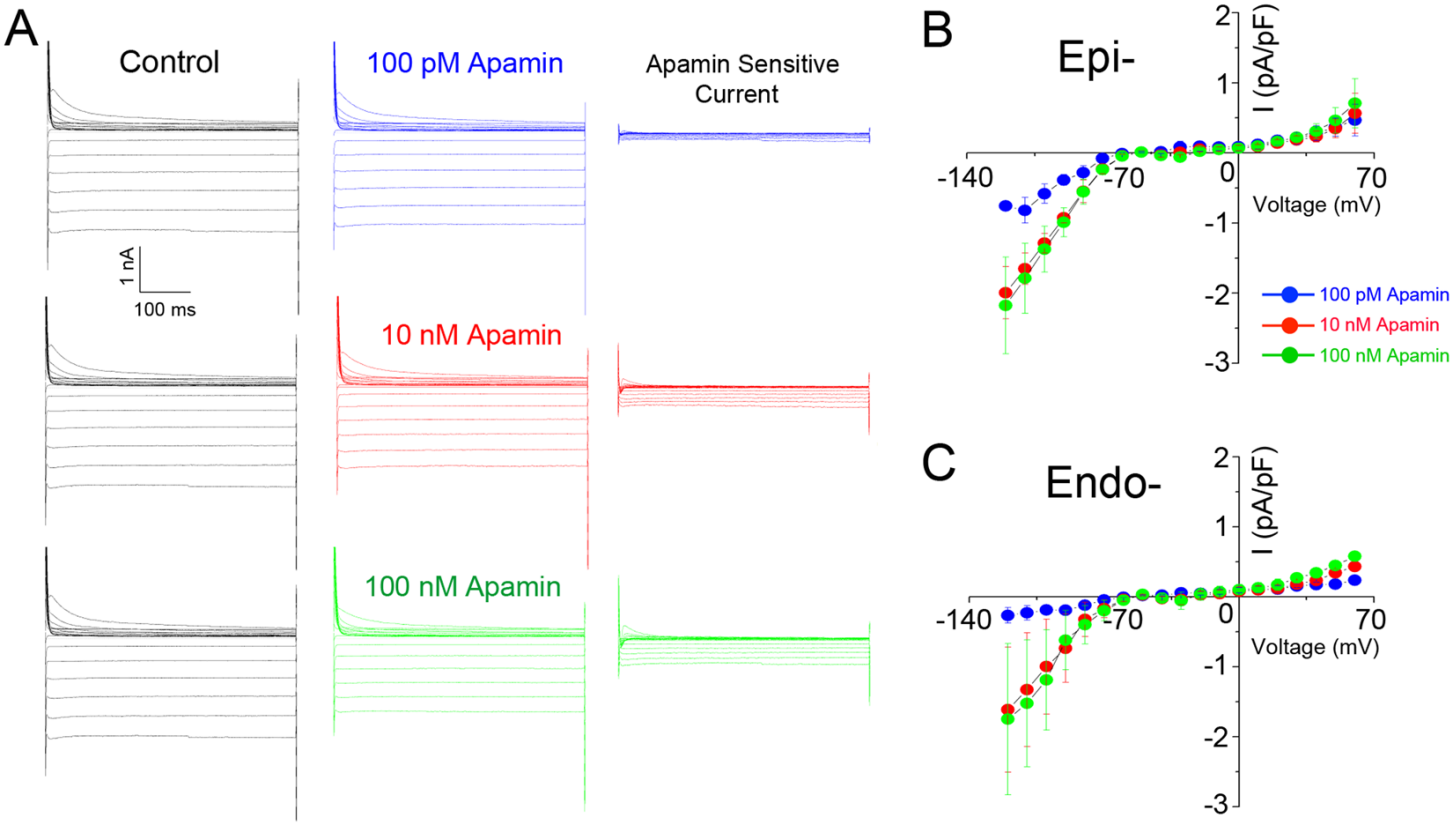
Apamin-sensitive SK currents in rabbit ventricular myocytes. (**A**) Representative current traces elicited from a holding potential of −55 mV using a family of voltage steps from −120 mV to +60 mV at 10 mV increments with 500 ms durations. Current traces shown are recorded in control (black traces, left panels), after 100 pM (blue traces), 10 nM (red traces), and 100 nM (green traces) of apamin (middle panels). Current traces in the right panels are apamin-sensitive currents obtained from digital subtraction. (**B** and **C**) Summary data for the current-voltage relationship of apamin-sensitive SK currents recorded from epicardial (Epi-) (n = 5) and endocardial (Endo-) (n = 7) ventricular myocytes.

Apamin-sensitive SK currents were previously identified in mouse and human hearts to play critical roles in atrial repolarization^3^. Recent studies further show that apamin-sensitive currents are expressed in rabbit ventricular myocytes^9,17^. Here, we document the functional expression of SK channels in rabbit ventricular myocytes by directly recording the apamin-sensitive currents and clamping the intracellular free Ca^2+^ concentration. Indeed, a recent study has shown that apamin is highly specific for SK channels^18^. Shown in Fig. 1A are representative apamin-sensitive currents using different concentrations of apamin. The corresponding current-voltage relations of the apamin-sensitive currents from epicardial and endocardial myocytes are shown in Fig. 1B,C, respectively. Our data support the functional expression of SK channels in rabbit ventricular myocytes.

### Isolation of SK currents activated by Ca^2+^ influx through LTCCs

**Figure 2:**
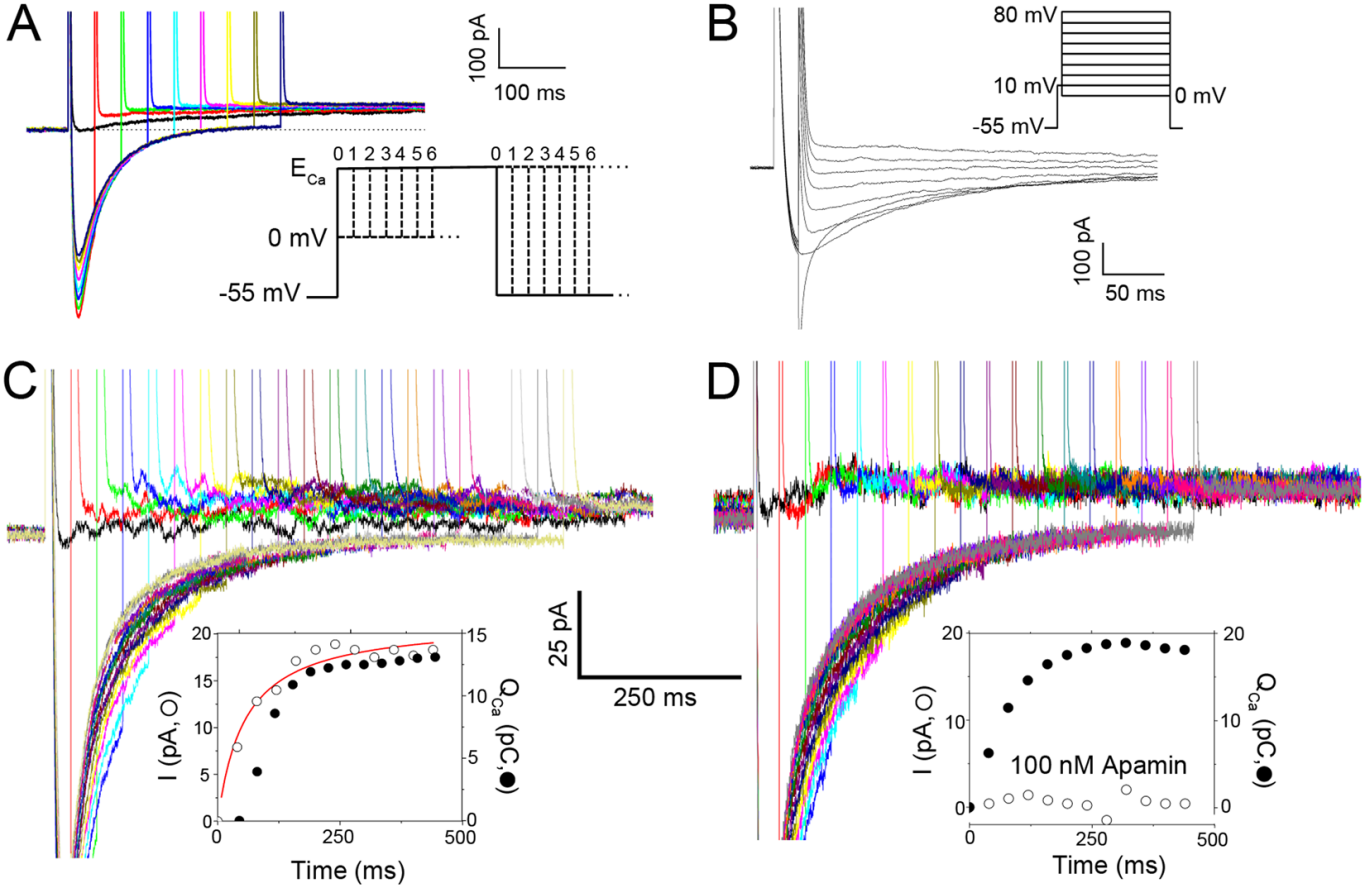
Apamin-sensitive outward currents elicited using two-pulse voltage-clamp protocols. (**A**) Representative current traces elicited from a two-pulse voltage-clamp protocol used to isolate apamin-sensitive current activated by Ca^2+^ influx through L-type Ca^2+^ channels (LTCCs). 50 μM BAPTA was included in the pipette solution. A diagram depicting the two-pulse voltage-clamp protocol is shown in the inset. (**B**) Current traces elicited using a voltage-clamp protocol to elicit Ca^2+^ currents for the determination of *E*_Ca_. A diagram depicting the voltage-clamp protocol is shown in the inset. (**C** and **D**) The outward SK currents in the absence (**C**) and presence of 100 nM apamin (**D**). The SK currents were activated by Ca^2+^ influx through LTCCs under conditions with 50 μM BAPTA in the pipette solution using the protocol shown in (**A**). The insets show activation kinetics of the outward K^+^ currents (in pA, open symbols) compared to the total charge entered through LTCCs during the prepulse (Q_Ca_ in pC, closed symbols).

To quantify SK currents activated by Ca^2+^ influx through LTCCs, we designed a voltage-clamp protocol using a prepulse to progressively induce Ca^2+^ influx *via* LTCCs, followed immediately by a test pulse to monitor the SK currents. To avoid contaminations from other currents, specific inhibitors for transient outward K^+^ current (*I*_to_), rapidly activating (*I*_Kr_) and slowly activating delayed rectifier K^+^ currents (*I*_Ks_), inward rectifier K^+^ currents (*I*_K1_), and Cl^−^ currents were applied during the recordings. In addition, the test pulse was stepped to the observed reversal potential for Ca^2+^ currents (*E*_Ca_) to minimize inward Ca^2+^ current (*I*_Ca_) during the test pulse. Figure 2A shows representative current traces with the voltage-clamp protocol shown as the inset. The instantaneous outward K^+^ currents progressively increased depending on the Ca^2+^ influx, suggesting intracellular Ca^2+^-dependence of the outward currents. For each cell, we first recorded the Ca^2+^ currents and measured *E*_Ca_, then applied the same potential as the test pulse to monitor the SK currents. The protocol for *I*_Ca_ recordings and the typical current traces are shown in Fig. 2B. To verify that the recorded currents represent SK currents, apamin was used. Experiments were performed using 50 μM of BAPTA in the pipette solution. The activation kinetics of the outward currents was directly quantified compared to the total charge entered through LTCC during the prepulse (Q_Ca_ in pC) (Fig. 2C). The outward currents could be completely blocked in the presence of 100 nM apamin as shown in the insets for Fig. 2D.

### Effects of BAPTA and EGTA on the activation of SK currents

**Figure 3:**
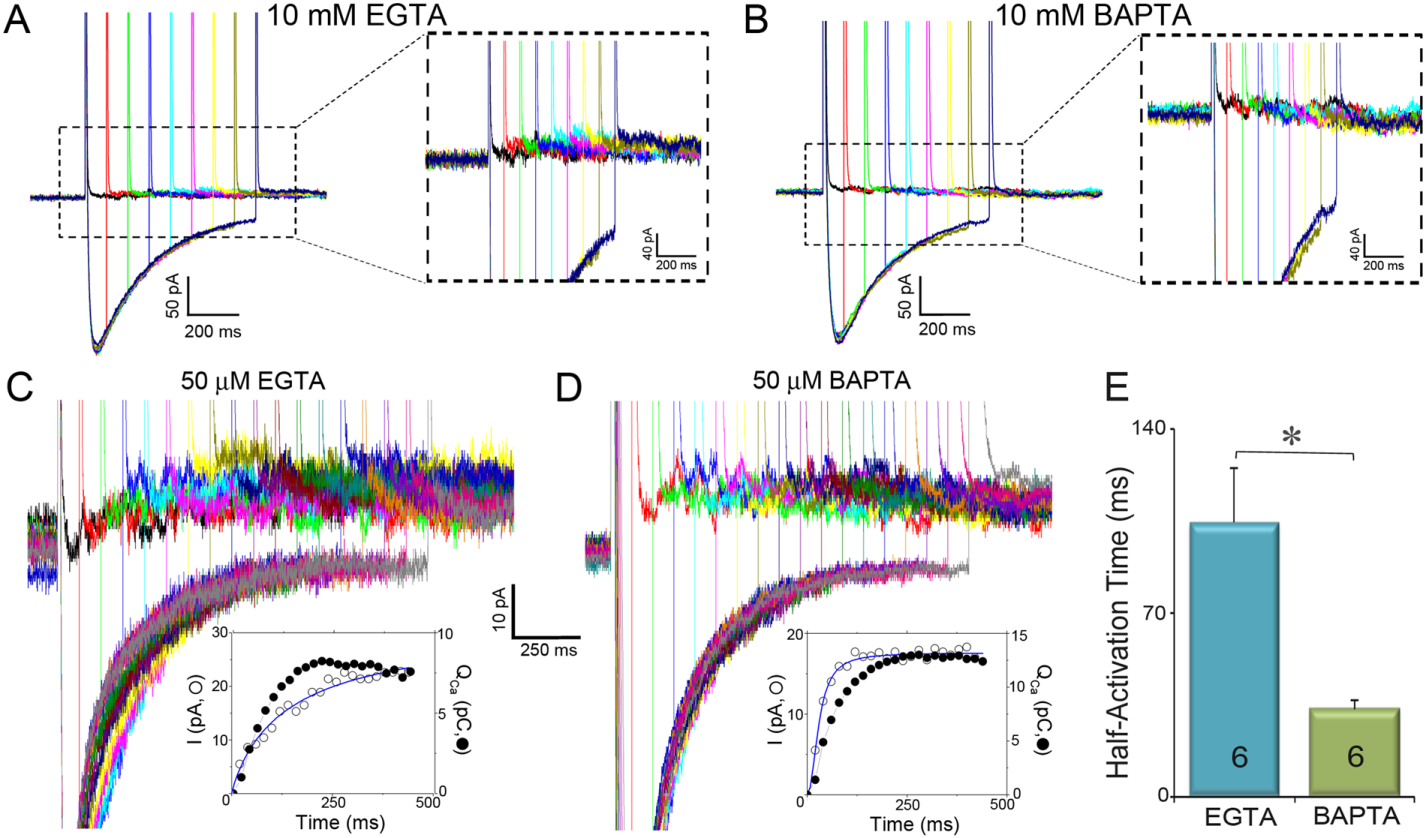
Effects of BAPTA compared to EGTA on the activation of SK currents. (**A** and **B**) Ca^2+^ influx through LTCCs does not activate SK currents when the pipette solution contains 10 mM EGTA (**A**) or 10 mM BAPTA (**B**). (**C** and **D**). SK currents activated by Ca^2+^ influx through LTCCs when 50 µM EGTA (**C**) or 50 µM BAPTA (**D**) was included in the pipette solution. The insets in (**C** and **D**) show activation kinetics of the outward K^+^ currents (in pA, open symbols) compared to the total charge entered through LTCCs during the prepulse (Q_Ca_ in pC, closed symbols). (**E**) Summary data of the half-activation time compared between 50 µM EGTA and 50 µM BAPTA as Ca^2+^ chelators (*P < 0.05).

BAPTA and EGTA are Ca^2+^ chelators with similar steady-state binding affinities but different binding rate constants with BAPTA being ~150 times faster than EGTA^19^. Therefore, the free Ca^2+^ concentration profile at the internal mouth of LTCCs is predicted to decline much more steeply with distance in BAPTA compared to EGTA^16^. In addition, previous studies have documented that processes that are sensitive to both BAPTA and EGTA are located within Ca^2+^ microdomains with distances ~50 nm to a few hundred nm. In contrast, processes that are affected by BAPTA, but not EGTA, are localized within “Ca^2+^ nanodomains” (within ∼20–50 nm of the Ca^2+^ source)^16,19^. We, therefore, compared the effects of BAPTA and EGTA on the activation of SK currents. Intracellular EGTA and BAPTA at a high concentration (10 mM) significantly reduced and completely abolished the Ca^2+^ influx-dependent, apamin-sensitive and progressively increasing K^+^ currents, respectively (Fig. 3A,B). The effects of both BAPTA and EGTA on the activation of SK currents suggest that SK channel lies within microdomains of its Ca^2+^ source.

Next, we tested the effects of intracellular BAPTA and EGTA at a lower concentration (50 µM) as shown in Fig. 3C–E. Here, the activation kinetics of the outward currents was directly quantified compared to the total charge entered through LTCCs during the prepulse (Q_Ca_ in pC). We observed the Ca^2+^ influx-dependent, apamin-sensitive and progressive increase in K^+^ currents in both buffer conditions. However, the activation kinetics of the SK currents was markedly different, with much faster activation time course when BAPTA was used compared to EGTA. The half-activation time was ~3–4 times smaller in BAPTA compared to EGTA.

### Activation of SK channels after depletion of SR Ca^2+^ store

**Figure 4:**
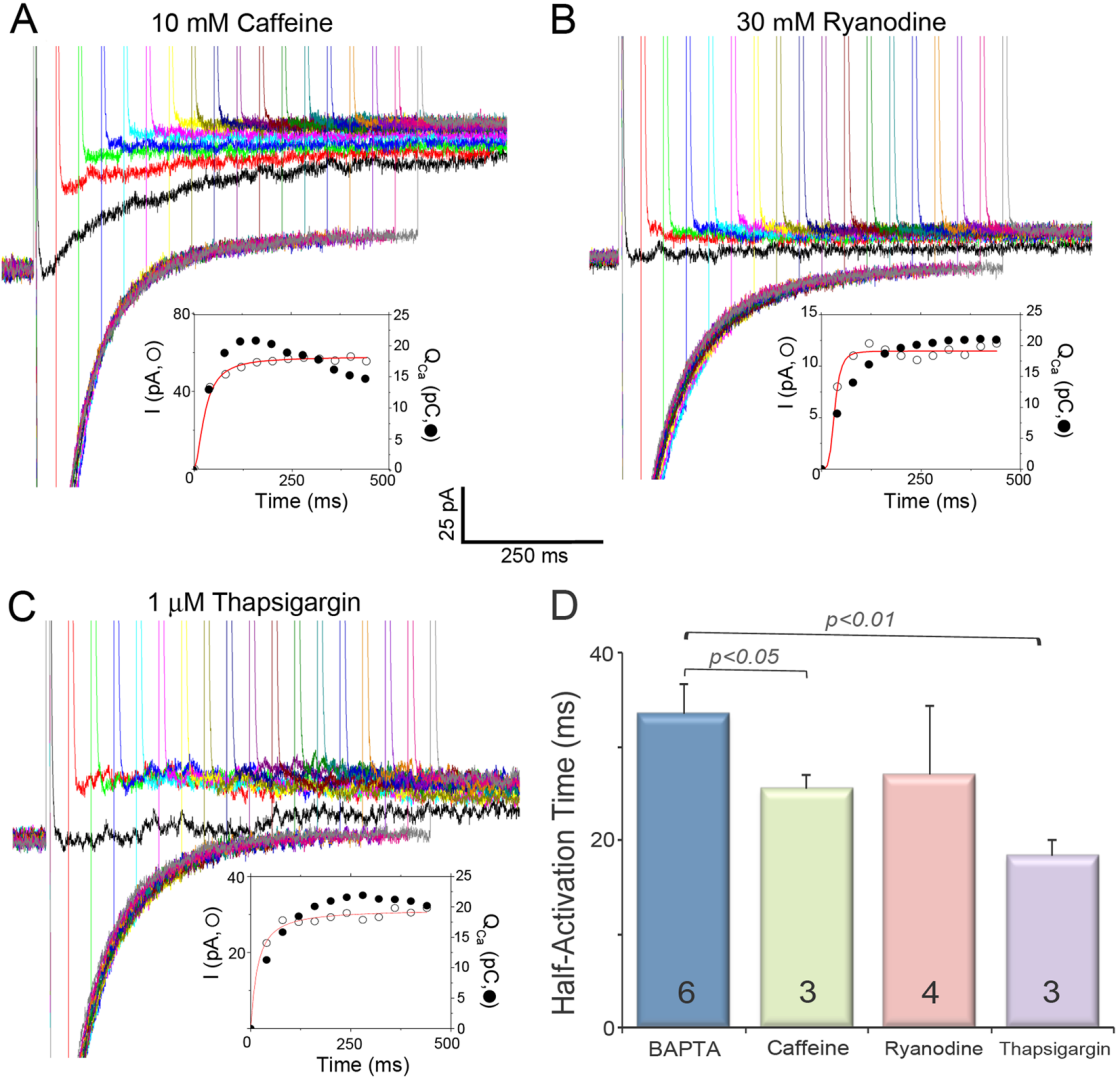
Activation of SK channels after depletion of the SR Ca^2+^ store. Activation of SK currents by Ca^2+^ influx through LTCCs in the presence of caffeine (**A**), ryanodine (**B**) and thapsigargin (**C**). The pipette solution contained 50 µM BAPTA. The insets in (**A–C**) show activation kinetics of the outward K^+^ currents (in pA, open symbols) compared to the total charge entered through LTCCs during the prepulse (Q_Ca_ in pC, closed symbols). (**D**) Summary data for the half-activation time under different conditions.

The Ca^2+^ influx through Ca^2+^ channels may also trigger Ca^2+^ release from the SR. Therefore, Ca^2+^ sources for the activation of the SK currents may originate from both Ca^2+^ influx through LTCCs and Ca^2+^ release from RyR2. To identify whether Ca^2+^ influx through LTCCs is sufficient to activate the SK channels, we inhibited the RyR2 Ca^2+^ release using 30 µM ryanodine or depleted the SR Ca^2+^ store by using 10 mM caffeine or 1 µM thapsigargin. As shown in Fig. 4A–C, SK currents were elicited even after the inhibition of the SR Ca^2+^ release, suggesting the critical role of Ca^2+^ influx through LTCCs in the activation of cardiac SK channels. However, the activation kinetics was significantly altered with shorter half-activation time when caffeine or thapsigargin was applied, suggesting the participation of SR Ca^2+^ release in the activation of SK channels (Fig. 4D). This hypothesis was further tested using high resolution imaging.

### Co-localization and quantification of SK2 channels with RyR2 and Ca_v_1.2 channels in rabbit ventricular myocytes

**Figure 5:**
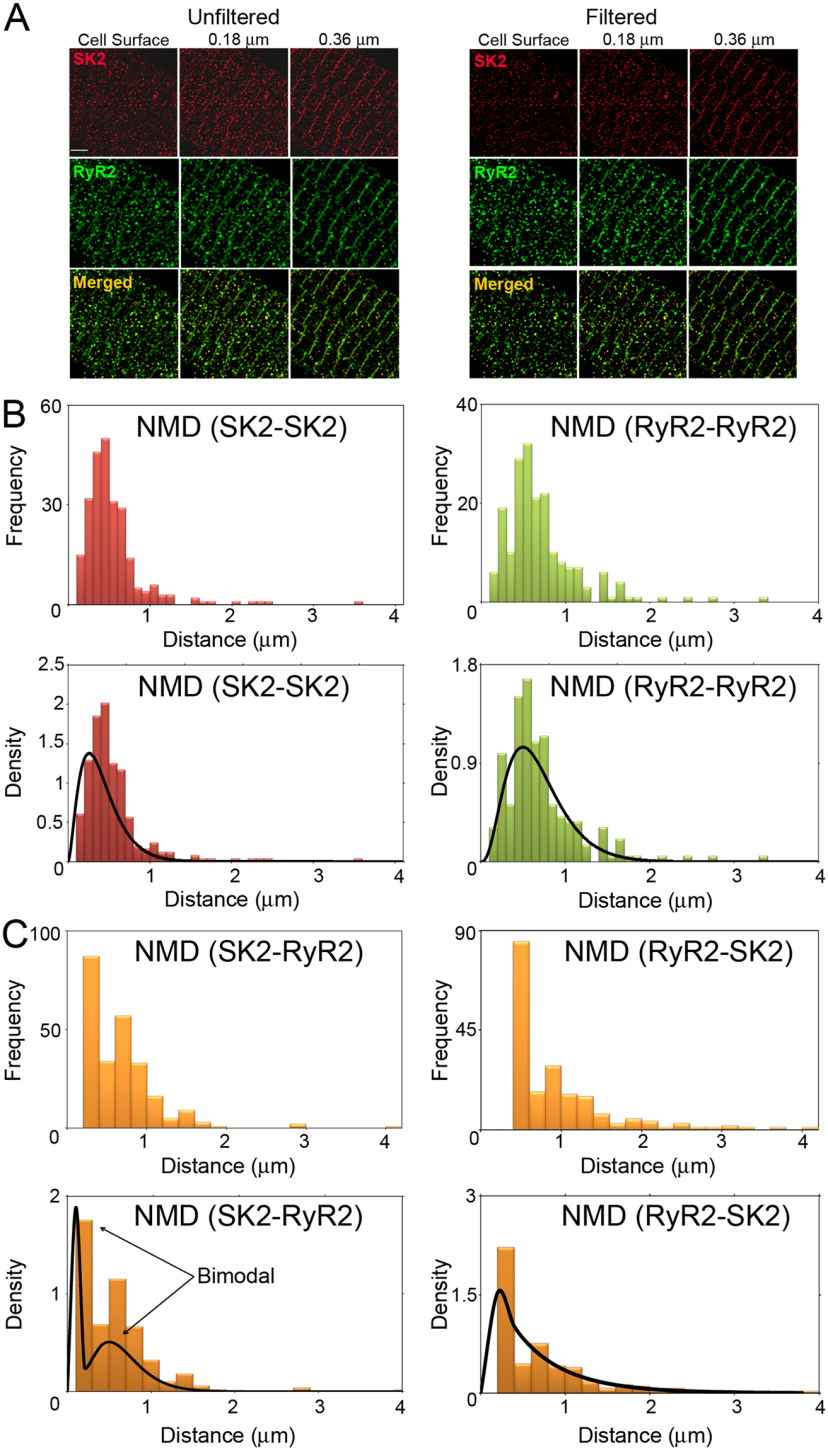
Spatial coupling of SK2 and RyR2 channels. (**A**) STED images of SK2 and RyR2 expression in rabbit ventricular myocytes. Unfiltered and filtered STED images at three Z planes (at the cell surface, 0.18 μm, and 0.36 μm from the cell surface) of SK2, RyR2, and merged images of the SK2 and RyR2. (**B**) Histograms depicting the frequency and density plots of NND for SK2 and RyR2 (NND(*SK2-SK2*) and NND(*RyR2-RyR2*)). (**C**) Histograms depicting the frequency and density plots of NND(*SK2-RyR2*) and NND(*RyR2-SK2*).

Figure 5A shows STED images of SK2 and RyR2 channel expression together with merged images in rabbit ventricular myocytes. Similar to SK2 expression in mouse cardiomyocytes^3^, SK2 channels are expressed along the Z lines as well as on the cell surface. We examined the co-localization of SK2 with RyR2 and Ca_v_1.2, a dominant isoform of LTCCs in ventricular myocytes, by quantifying the distance based on the Z-stack STED images. The localization of the channels was analyzed in two distinct regions: on the surface and in the interior of the cell (i.e. from Z planes located at the cell surface, 0.18, and 0.36 µm from the surface).

Results shown are obtained from first filtering and thresholding the STED images to detect channel clusters. Figure 5B,C show each step involved in determining the nearest neighbor distances (NND). For clarity, we introduce the following notation: let NND(*X-Y*) denote the nearest neighbor distances between channel *X* and channel *Y*. That is, NND(*SK*2*-RyR2*) will denote the NND between SK2 and RyR2 channels. Since NDD(*SK2-RyR2*) may differ from NND(*RyR2-SK2*), we will report both findings and determine if there is a significant difference between these two measurements.

### SK2 and RyR2 channels

The distributions pertaining to SK2 and RyR2 channels are summarized in Fig. 5B. Both NND(*SK2-SK2*) and NND(*RyR2-RyR2*) follow a unimodal Gamma distribution. NND(*SK2-SK2*) are 0.57 ± 0.51 μm while NND(*RyR2-RyR2*) are 0.83 ± 0.54 μm (data represent mean ± standard deviation). Indeed, the measurement for RyR2 channels from STED images closely agrees with the previous measurements using confocal imaging^20^ and super-resolution technique based on single-molecule localization^21^.

The inter-channel (*RyR2-SK2* and *SK2-RyR2*) NNDs are summarized in Fig. 5C. There was a statistically significant difference between NND(*RyR2-SK2*) and NND(*SK2-RyR2*). This difference can be explained by the fact that we observed more RyR2 than SK2 channels. NND(*SK2-RyR2*) are 0.47 ± 0.46 μm while NND(*RyR2-SK2*) are 0.55 ± 0.65 μm. Importantly, as shown in Fig. 5C, NND(*SK2-RyR2*) is bimodal with first and second modes at 0.09 μm and 0.41 μm, respectively. This bimodal distribution can be obtained from a finite mixture of two Gamma distributions. The bimodal aspect of NND(*SK2-RyR2*), as opposed to an exponential distribution, suggests a relationship (either functional or spatial) between the two channels.

Our analyses further suggest that there is a statistical difference in the distribution of the two channels localized on the surface (Table 1) or in the interior of the cells (Table 2). For channels localized on the cell surface, NND(*SK2-SK2*) are 0.50 ± 0.23 μm while NND(*RyR2-RyR2*) are 0.62 ± 0.27 μm. NND(*RyR2-SK2*) and NND (*SK2-RyR2*) are 0.29 ± 0.31 μm and 0.29 ± 0.28 μm, respectively (*P* = NS).

**Table 1:** Mean and standard deviations of the nearest neighbor distances from one type of channel to the other type of channel on the cell surface.

Surface nearest neighbor distances (μm)		
From\To	RyR2	SK2
RyR2	0.62 ± 0.27	0.29 ± 0.31^§^
SK2	0.29 ± 0.28^§^	0.50 ± 0.23^¶^

^¶^Denotes significant difference for NND(SK2-SK2) between the surface and in the interior of the cells, *P* < 0.05.

^§^Denotes significant difference for NND(RyR2-SK2) and NND(SK2-RyR2) between the surface and in the interior of the cells, *P* < 0.05.

**Table 2:** Mean and standard deviations of the nearest neighbor distances from one type of channel to the other type of channel in the cell interior.

Interior nearest neighbor distances (μm)		
From\To	RyR2	SK2
RyR2	0.63 ± 0.28	0.76 ± 0.55*^§^
SK2	0.48 ± 0.36*^§^	0.68 ± 0.53^¶^

*Denotes significant difference between NND(RyR2-SK2) and NND(SK2-RyR2), *P* < 0.05.

^¶^Denotes significant difference for NND(SK2-SK2) between the surface and in the interior of the cells, *P* < 0.05.

^§^Denotes significant difference for NND(RyR2-SK2) and NND(SK2-RyR2) between the surface and in the interior of the cells, *P* < 0.05.

For channels localized within the interior of the cells, NND(*SK2-SK2*) are 0.68 ± 0.53 μm while NND(*RyR2-RyR2*) are 0.63 ± 0.28 μm. In contrast to the cell surface, there was a statistically significant difference between NND(*RyR2-SK2*) and NND(*SK2-RyR2*) (0.76 ± 0.55 and 0.48 ± 0.36 μm, respectively) for channels localized within the cell interior (*P* < 0.05).

There were no statistically significant differences between the NND of RyR2 channels distributed on the cell surface or the interior of the cells. There was, however, a statistically significant difference in SK2 channel distributions between cell surface and the interior (Tables 1 and 2). In addition, there was a statistically significant difference between NND from RyR2 to SK2 and from SK2 to RyR2 on the surface and in the interior of the cells.

Colocalization analyses using Volocity yields Pearson’s correlation coefficients of 0.62 and 0.44 between RyR2 and SK2 channels localized on the cell surface compared to the cell interior, respectively. These correlation coefficients provide a measure of colocalization based on the spatial distribution of color intensities in a 2D or 3D image, and can range in value from −1 (negative linear correlation) to +1 (positive linear correlation).

Another measure of colocalization that has the benefit of being an intuitive interpretation is the probability of the presence of one channel given that there is record of another. These probabilities simply show how likely we are to see one particular channel if we are certain (or there is a record) of the presence of another channel. We calculate the conditional probabilities based on the presence of signals rather than their intensities (please see additional details in Materials and Methods). On the cell surface, the probability of the presence of an RyR2 channel given that there is record of an SK2 channel (Prob (RyR2|SK2)) was 67.8% while the probability of the presence of an SK2 channel given that there is a record of an RyR2 channel (Prob (SK2|RyR2)) was 56.3%. In the cell interior, Prob (RyR2|SK2) and Prob (SK2|RyR2) were 54.1 and 53.6%, respectively. In summary, ~2/3 of RyR2 channels have associated SK2 channels while ~half of SK2 channels have associated RyR2 channels.

### SK2 and Ca_v_1.2 channels

**Figure 6:**
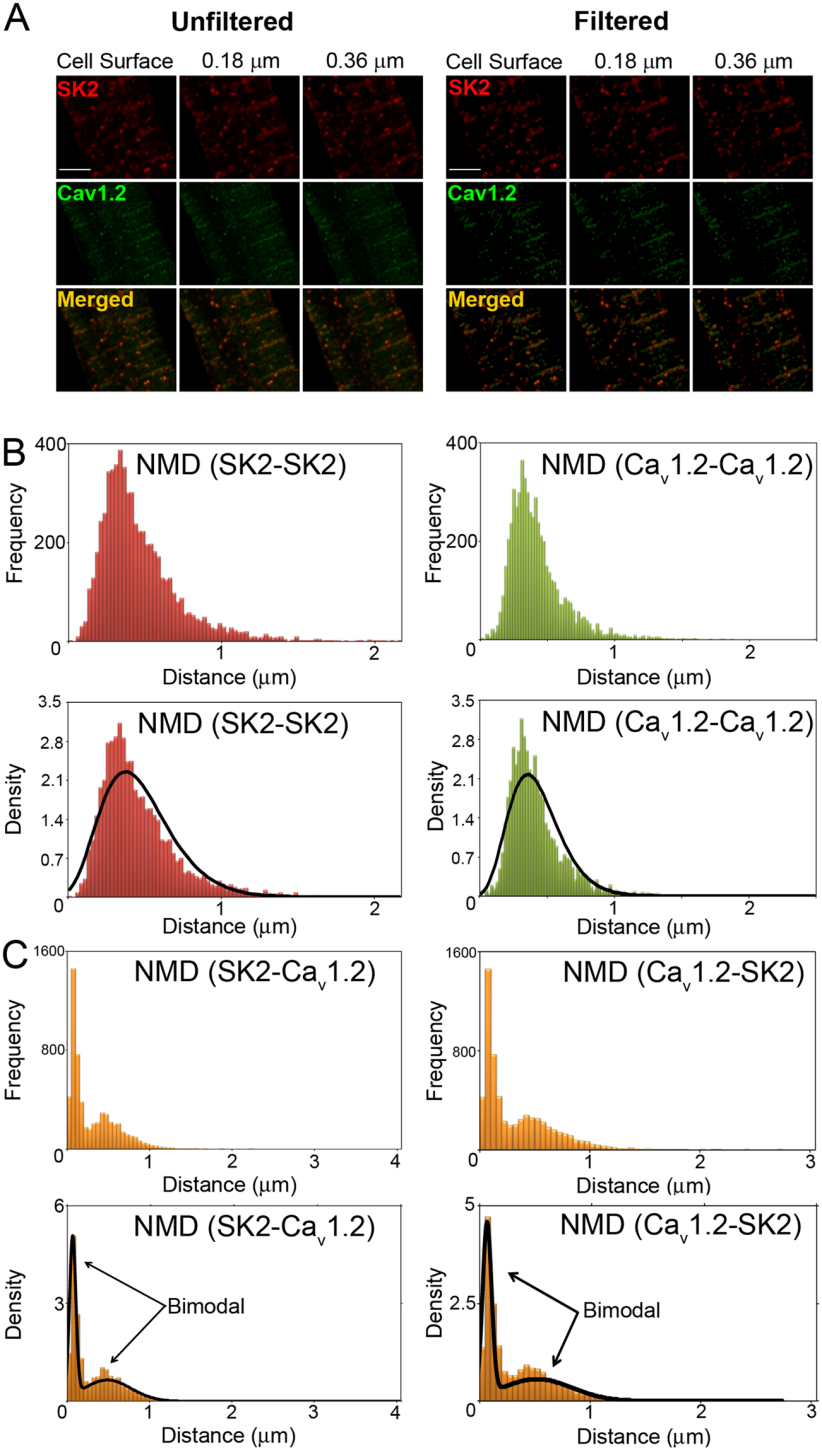
Spatial coupling of SK2 and Ca_v_1.2 channels. (**A**) STED images of SK2 and Ca_v_1.2 expression in rabbit ventricular myocytes. Unfiltered and filtered STED images of the three Z planes (as in Fig. 5) of SK2 channels, Ca_v_1.2, and merged images of the SK2 and Ca_v_1.2. (**B**) Histograms depicting the frequency and density plots of NND for SK2 and Ca_v_1.2 (NND(*SK2-SK2*) and NND(*Ca*_*v*_*1.2-Ca*_*v*_*1.2*). (**C**) Histograms depicting the frequency and density plots of NND(*SK2- Ca*_*v*_*1.2*) and NND(*Ca*_*v*_*1.2-SK2*).

Figure 6A shows STED images of SK2 and Ca_v_1.2 channel expression together with merged images in rabbit ventricular myocytes. NND(*SK2-SK2*) and NND(*Ca*_*v*_*1.2-Ca*_*v*_*1.2*) are summarized in Fig. 6B. Both NND(*SK2-SK2*) and NND(*Ca*_*v*_*1.2-Ca*_*v*_*1.2*) follow a unimodal Gamma distribution. The inter-channel (*SK2-Ca*_*v*_*1.2* and Ca_v_1.2-*SK2*) NNDs are summarized in Fig. 6C & Table 3. There were no significant differences between inter-channels distributions for NND(*SK2-Ca*_*v*_*1.2*) and NND(*Ca*_*v*_*1.2-RyR2*). Notice that NND(*SK2-Ca*_*v*_*1.2*) displays the characteristics of a bimodal distribution with first and second modes at 0.08 and 0.41 μm, respectively. This bimodal distribution can be obtained from a finite mixture of two Gamma distributions. Similar to SK2 and RyR2 channels, the bimodal distribution of NND (as opposed to an exponential distribution) suggests that there is a non-random functional or spatial relationship between SK2 and Ca_v_1.2 channels. Additionally, the Pearson’s correlation coefficient between SK2 and Ca_v_1.2 was calculated to be 0.8. This is a strong indication that SK2 and Ca_v_1.2 channels co-localize.

**Table 3:** Summary of NNDs of SK2 and Ca_v_1.2 (in μm).

From\To	Ca_v_1.2	SK2
Ca_v_1.2	0.54 ± 0.21	0.31 ± 0.29
SK2	0.34 ± 0.32	0.49 ± 0.21

## Discussion

SK channels contribute to cardiac repolarization, participate in electrical remodeling in heart failure and atrial fibrillation, and may serve as potential therapeutic targets against cardiac arrhythmias^4–7^. A decade of studies of cardiac SK channels have paved the way for the mechanistic understanding of the critical roles of SK channels in the regulation of cardiac excitability^4^. However, the exact mechanistic underpinning for the activation of cardiac SK channels remains a key but unaddressed question in the field. The unique gating property of SK channels solely by intracellular Ca^2+^ highlights the significance of Ca^2+^ signaling in the regulation of SK channels. Two central molecules dominating the intracellular Ca^2+^ signaling in cardiomyocytes are LTCC and RyR2. Therefore, understanding the physical and functional relationships between these two molecules and SK channels is important not only from biophysical but also physiological and pathological points of view.

Our previous study reported that cardiac SK2 channels interact with LTCCs through a physical bridge, α-actinin2^13^. The interaction localizes SK channels to the entry of external Ca^2+^ source, suggesting the functional coupling between cardiac SK2 and LTCCs. SK channels are very sensitive to local subsarcolemmal Ca^2+^ and are activated by submicromolar concentrations of intracellular Ca^2+^ ions with apparent *K*_d_ of ~0.5 µM^2^. Our current study provides the estimation of the physical distance of LTCCs and RyR2 within hundreds of nanometers from SK2 channels in cardiomyocytes. The spatial proximity of the three molecules enables the optimal activation and precise control of the SK2 gating process on the beat-to-beat basis by integrating the local Ca^2+^ signaling. Moreover, SK channel activation is predicted to provide a feedback mechanism to regulate the activities of LTCCs and RyR2 to influence the local and global Ca^2+^ signaling through the effects of SK channels on membrane potentials.

There are three subtypes of SK channels, SK1, SK2, and SK3, encoded by three distinct genes, *KCNN1*, *2*, and 3. The three subtypes of SK channels differ in their sensitivity to apamin^1^. All three subtypes are expressed in rabbit ventricular myocytes, as supported by the current recordings when three different concentrations of apamin were applied (Fig. 1). Since they are gated by similar submicromolar Ca^2+^, our current study does not differentiate the coupling mechanisms of the different subtypes. Further studies are necessary to differentiate the contributions from different subtypes of SK channels.

### Regulation of subsarcolemmal Ca^2+^ concentration in cardiomyocytes

The regulation of subsarcolemmal Ca^2+^ concentration is a dynamic process orchestrated by the activities of LTCCs, RyR2, SR Ca^2+^-ATPase, sarcolemmal Na^+^/Ca^2+^ exchange, sarcolemmal Ca^2+^-ATPase, and mitochondrial Ca^2+^ uniporter. Among all the molecules involved in the process, LTCCs play a central role in triggering the subsarcolemmal Ca^2+^ increase leading to Ca^2+^ release from the SR through RyR2. The Ca^2+^ influx through LTCCs raises the local concentration from 0.1 to 10 µM, and subsequent Ca^2+^ release through RyR2 further increases the cleft Ca^2+^ concentration to ~100 µM, even though the global intracellular Ca^2+^ concentration only reaches ~1 µM^12,22^. Activation of cardiac SK channels is predicted to be tightly controlled by the temporal and spatial Ca^2+^ signaling due to the strategic location of SK channels within the t-tubules.

SK channels have also been reported to be expressed in the inner mitochondrial membrane and play a protective role against ischemia/reperfusion injury by enhancing the matrix K^+^ entry and inducing a preconditioning effect^23^. A recent report further revealed that activation of mitochondrial SK channels attenuates Ca^2+^-dependent arrhythmia in hypertrophic hearts by reducing the production of mitochondrial reactive oxygen species and subsequent oxidation of RyR2 channels^24^. Therefore, mitochondrial SK channels contribute to intracellular Ca^2+^ homeostasis which may affect the activation of SK channels on the plasma membrane. However, the activation mechanism of mitochondrial inner membrane SK channels remains incompletely understood. Nonetheless, the SK currents recorded in our study are directly attributed to the plasma membrane SK channels.

### Distinct effects of BAPTA compared to EGTA

Local Ca^2+^ signaling can be assessed using Ca^2+^ chelators, EGTA and BAPTA^25,26^. Both EGTA and BAPTA have similar steady-state binding affinities for Ca^2+^, however, the binding rate constants of BAPTA are about 150 times faster than EGTA^19^. Therefore, BAPTA is a more effective chelator in preventing the diffusion of free Ca^2+^ away from the source. The cytoplasmic Ca^2+^ concentration profiles at the inner mouth of LTCCs are predicted to show a steeper decline with distance in the presence of BAPTA compared to EGTA as the intracellular Ca^2+^ buffers^16^. As such, the local Ca^2+^ signaling domains at the inner mouth of LTCCs were classified as Ca^2+^ nanodomains and microdomains based on the different effects of intracellular EGTA and BAPTA on the Ca^2+^-dependent cellular processes^25,26^.

The coupling between SK channels and LTCCs was first studied in dissociated hippocampal pyramidal neurons^27^. Cell-attached single channel recordings showed the depolarization-induced brief inward Ca^2+^ currents followed by outward SK currents. Due to the intrinsically high Ca^2+^ sensitivities of SK channels, the distance between the internal mouth of LTCCs and SK channel activation sites may be tens of nanometers to a hundred of nanometers, which remains adequate to provide saturating concentrations of Ca^2+^ for the activation of SK channels. Based on the intracellular Ca^2+^ concentration profiles with EGTA/BAPTA and the Ca^2+^ sensitivity of SK channels, a 20–100 nm distance was estimated between SK channels and the inner mouth of LTCCs in hippocampal pyramidal neurons^16^. The findings that BAPTA reduced the coupling between LTCCs and SK channels in hippocampal pyramidal neurons further strengthened the estimation^27^.

In the current study, we found that 10 mM BAPTA and EGTA significantly disrupt the coupling between cardiac SK channels and LTCCs suggesting SK channels are located within the microdomain of the Ca^2+^ source generated by LTCCs in cardiomyocytes. Our high resolution STED images and the quantitative analysis of the images further support the spatial proximity of the two channels in cardiomyocytes. Using lower concentrations of intracellular BAPTA or EGTA, we were able to elicit the apamin-sensitive SK currents activated by Ca^2+^ influx through LTCCs. However, the activation kinetics with BAPTA as the intracellular Ca^2+^ buffer was significantly faster than that of EGTA. This may result from the ability of Ca^2+^-bound BAPTA behaving as a mobile buffer to enhance the spatial diffusion of Ca^2+^ ^19,28^. Single channel recordings in hippocampal pyramidal neurons demonstrated that BAPTA-treated patches have prolonged LTCC openings^27^. The overall effects of BAPTA result from its ability to reduce the steady-state level of inactivation and increase the rate of recovery from inactivation of LTCCs^29^.

### Insight into the spatial distribution of SK2, RyR2 and Ca_v_1.2 channels

Our analysis of the nearest neighbor distributions for SK2-Ca_v_1.2 and SK2-RyR2 display the characteristics of a bimodal distribution. This bimodal distribution can be obtained from a finite mixture of two Gamma distributions. The bimodal distribution of NND (as opposed to an exponential distribution) suggests that there is a non-random functional or spatial relationship between SK2 and RyR2, as well as SK2 and Ca_v_1.2 channels. Additionally, the Pearson’s correlation coefficient between SK2 and Ca_v_1.2 was calculated to be 0.8. This is a strong indication that SK2 and Ca_v_1.2 channels co-localize.

**Figure 7:**
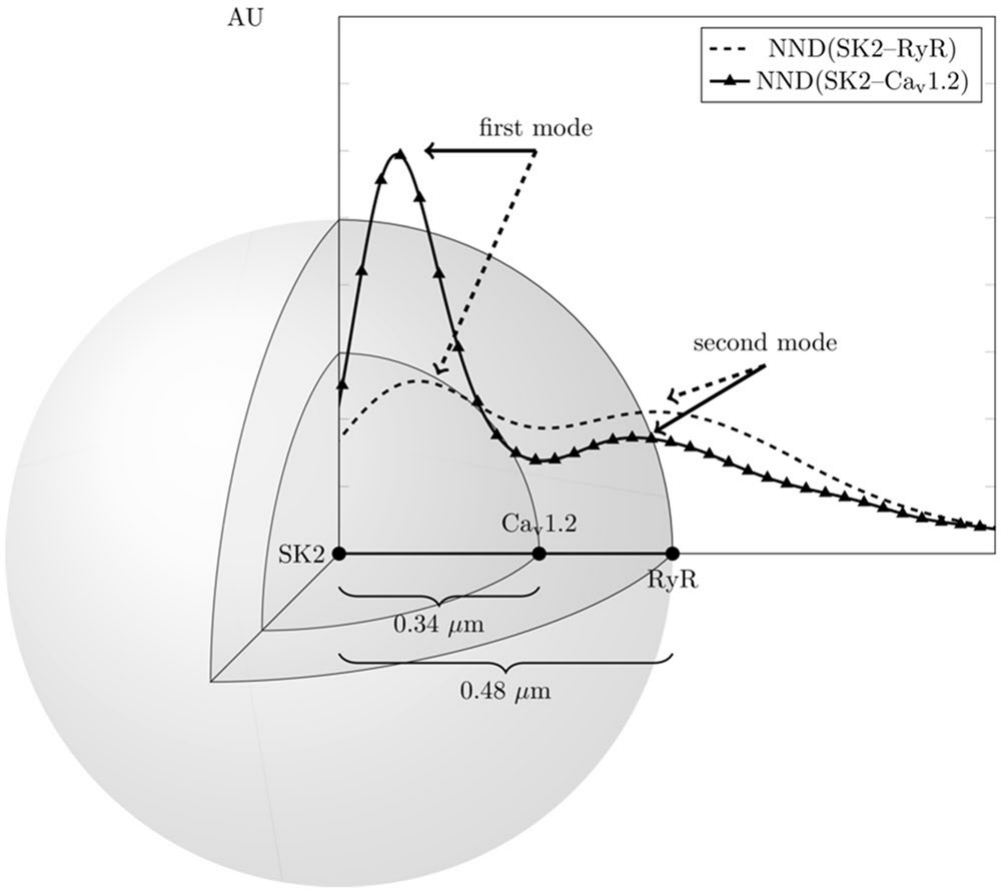
Visualization of the spatial distribution of SK2, RyR2, and Ca_v_1.2 (AU: arbitrary units).

Figure 7 summarizes the spatial distributions of these three molecules with SK2 being at the center of the relationship. The qualitative similarity of NND(*SK2-RyR2*) and NND(*SK2-Ca*_*v*_*1.2*) is consistent with the well documented co-localization between Ca_v_1.2 and RyR2 channels. Indeed, both the first and second modes of the two distributions are within 0.01 μm of one another.

### Functional units of SK2, Ca_v_1.2, and RyR2 channels

Using SK2 channel-overexpressing adult rat ventricular myocytes, it was reported that the complete depletion of SR Ca^2+^ content eliminated SK current in response to depolarization with intact Ca^2+^ influx through LTCCs, suggesting that SR Ca^2+^ release is both necessary and sufficient for the activation of SK channels in rat ventricular myocytes^14^. However, overexpression of SK2 channels in rat ventricular myocytes may significantly alter the SK2 localization in relations to LTCCs and RyR2. In contrast, our study explored the activation mechanism of endogenous SK channels in rabbit ventricular myocytes. Our findings revealed that Ca^2+^ influx through LTCCs provides the immediate and efficient Ca^2+^ microdomain for the activation of SK channels. Moreover, changes in SK channel activation kinetics following SR Ca^2+^ depletion suggest that both LTCC-mediated Ca^2+^ influx and SR Ca^2+^ release are necessary for the activation of cardiac SK channels. Indeed, our results are further supported by a recent study which shows that the inhibition of RyR2 or SERCA or knockdown of RyR2 by shRNA reduces but does not completely eliminate the SK currents in mouse atrial myocytes^15^.

To avoid the interference of ATP-sensitive currents, we did not include ATP in the pipette solution, which may affect the SR Ca^2+^ uptake and release. Nonetheless, in our recording condition, SK channel activation kinetics was significantly altered after the inhibition of the SR Ca^2+^ release, suggesting the important role of SR Ca^2+^ release in the activation of SK channels (Fig. 4). There are possibilities that the importance of SR Ca^2+^ release may be underestimated due to the absence of ATP in pipette solution, which will be further investigated in our future studies.

Since SK channels are gated solely by changes in intracellular Ca^2+^, the coupling of SK channels with LTCC and SR Ca^2+^ release provides an immediate feedback mechanism for the regulation of cardiac AP duration on a beat-to-beat basis, which in turn controls cardiac excitability, excitation-contraction coupling, and contractility. Moreover, the activation mechanism revealed by our study paves the way for the understanding of the roles of SK channels in arrhythmia- and heart failure-induced electrical remodeling as well as the possible roles of SK channels on the feedback mechanism to regulate the activities of LTCCs and RyR2 to influence the local and global Ca^2+^ signaling.

## Methods

### Isolation of ventricular myocytes from rabbit heart

All animal care and procedures were approved by UC Davis Institutional Animal Care and Use Committee and was in accordance with NIH and institutional guidelines. For isolation of the rabbit ventricular myocytes, New Zealand White rabbits (males, 3–4 months old, 2.5–3 kg) were first injected with heparin (5000 U) and then anesthetized with isoflurane (5%). After achieving deep anesthesia, a standard enzymatic technique was used to isolate ventricular myocytes at 37 °C. Briefly, hearts were mounted on a Langendorff system and retrogradely perfused for 5 min with oxygenated solution containing (in mmol/l): NaCl 138, KCl 5.4, CaCl_2_ 0.05, MgCl_2_ 1, NaH_2_PO_4_ 0.33, 10 NaHCO_3_, N-2-hydroxyethylpiperazine-N-2-ethane sulfonic acid (HEPES) 10, glucose 6, pyruvic acid 2.5, pH = 7.4. When blood was removed from the coronary circulation, the solution was supplemented with 1 mg/ml type II collagenase (305 U/mg; Worthington Biochemical Co., Lakewood, NJ, USA), 0.05 mg/ml protease type XIV (Sigma-Aldrich Co., St. Louis, MO, USA) and 1 mg/ml bovine serum albumin. The heart was perfused for 30 min to enzymatically dissociate cells. Portions of the left ventricular wall were cut into small pieces and the cell suspension was washed with the above solution. Finally, the Ca^2+^ concentration was gradually restored to 1.2 mM.

### Stimulated emission depletion (STED) imaging

STED imaging was performed using Leica TCS SP8 STED 3× microscope. Isolated rabbit ventricular myocytes were fixed with 4% paraformaldehyde on No. 1.5-thickness/12-mm-round glass coverslips (Thomas Scientific, Swedesboro, NJ, USA) for 20 minutes at room temperature (RT), and then washed 3 times with phosphate buffered saline (PBS) containing 0.05% Triton-X. Non-specific antibody binding was blocked with 10% bovine serum albumin (BSA) in 0.1% phosphate buffered saline with Tween 20 PBST for 1 hour at RT. For double labeling experiments, cells were first treated with a primary antibody (anti-SK2 polyclonal antibody (Abcam 111939, Cambridge, MA, USA), which was tested in HEK 293 cells expressing human cardiac SK2 (Supplemental Fig. 1); anti-Ca_v_1.2 monoclonal antibody (NeuroMab Cav1.2 N263/31, Davis, CA, USA)^30^; or anti-RyR2 monoclonal antibody (Thermo Fisher Scientific, Clone C3-33, Rockford, IL, USA))^31^, incubated overnight at 4 °C, followed by application of secondary antibodies sequentially with tetramethylrhodamine (TMR) donkey-anti-goat secondary antibody (Thermo Fisher Scientific) followed by Oregon Green 488 goat-anti-mouse (Thermo Fisher Scientific) for 1 hour incubation at RT. For STED imaging, ProLong Gold Antifade Mountant (Thermo Fisher Scientific) was used to minimize photobleaching. Negative controls with secondary antibodies alone were performed for all experiments. For testing the SK2 antibody, Human Embryonic Kidney (HEK) 293 cells were cultured and transfected by human cardiac SK2 plasmid using the similar methods we reported before^32^.

### STED image analysis

The analysis of STED images was based on the three-dimensional detection of SK2 channel and RyR2 clusters. The channel clusters were identified by thresholding the STED images consisting of 48 Z planes with a Z-step of 0.18 µm. The threshold value was obtained from an iterating process that is similar to the widely used spark detection method^33,34^. The colocalization of SK2, RyR2, and Ca_v_1.2 was analyzed using a software package Volocity with Quantitation (Perkin–Elmer, Waltham, MA) implementing the standard Pearson’s colocalization analysis^35^.

(A). Image filtering and channel cluster detection
  The channel clusters were detected by thresholding the pixel intensity of each image. The threshold value was obtained from a filtering process that is similar to the widely used spark detection method^33,34^ and can be summarized as follows. An image, *I*_1_, is generated from the raw image, *I*_0_, based on pixels with intensity lower than a criterion. The criterion is *μ*_0_ + *ασ*_0_, where *μ*_0_ and *σ*_0_ refer to the mean value and standard deviation of the raw image *I*_0_. A working and filtered image, *I*_w_, is constructed by applying a mask *M*_1_ to the raw image *I*_0_. The mask is a binary image at a level of *μ*_1_ + *βσ*_1_, where *μ*_1_ and *σ*_1_ are the mean value and standard deviation of the image *I*_1_, respectively. A threshold, *τ*_i_, is calculated by taking the ratio of the mean of the original image, *μ*_0_, to the mean, μ*, of the pixels with non-zero intensity of the working image.
  The threshold, *τ*, is calculated by taking the minimum value of all the thresholds, *τ*_i_, generated from all raw images. The threshold, *τ*, is then used in the Volocity 3D image analysis software (see http://www.perkinelmer.com/ for more details) to identify three-dimensional connected regions. The filtering process and the statistical calculations were performed in Python. In this study, we used the parameters *α* = 1 and *β* = 1.
(B). Conditional probabilities
  The conditional probabilities of SK2 given RyR2 and RyR2 given SK2 were calculated based on the presence of signals in the recordings as follows:
  1 $${\rm{Prob}}\,({\rm{RyR}}2|{\rm{SK}}2)=\frac{\sum ({\rm{Signal}}\,{\rm{Presence}}\,{\rm{for}}\,{\rm{SK}}2\times {\rm{Signal}}\,{\rm{Presence}}\,{\rm{for}}\,{\rm{RyR}}2)}{\sum ({\rm{Signal}}\,{\rm{Presence}}\,{\rm{for}}\,{\rm{SK}}2)}.$$
  2 $${\rm{Prob}}\,({\rm{SK}}2|{\rm{RyR}}2)=\frac{\sum ({\rm{Signal}}\,{\rm{Presence}}\,{\rm{for}}\,{\rm{SK}}2\times {\rm{Signal}}\,{\rm{Presence}}\,{\rm{for}}\,{\rm{RyR}}2)}{\sum ({\rm{Signal}}\,{\rm{Presence}}\,{\rm{for}}\,{\rm{RyR}}2)}$$
  where the signal presence for a channel is 1 when a signal is shown on the STED image and 0 otherwise. To remove the effect of noise, the conditional probabilities were computed using STED images filtered using the spark detection algorithm. Similar strategy was used for SK2 and Ca_v_1.2 analysis.
(C). Distributions and finite mixtures

The following notation is used. NND(*X-Y*) is used to denote the distribution of the nearest neighbor distances between channels *X* and *Y*. For example, NND (*SK2-SK2*) stands for the nearest neighbor distribution between SK2 clusters, while NND (*SK2-RyR2*) stands for the nearest neighbor distribution between SK2 and RyR2 clusters. All distributions were fitted using a finite mixture of Gamma distributions. The choice of a gamma distribution is based on the negative skewness of the data as well as its positive support. For a population of nearest neighbor distances, the following formula for finite mixture was used:

3 $$NND(X-Y)={\sum }_{(i=0)}^{K}{\lambda }_{i}{\rm{\Gamma }}({\alpha }_{i},\,{\beta }_{i})$$

where

$${\sum }_{(i=0)}^{K}{\lambda }_{i}=1,$$

with *α*_*i*_ and *β*_*i*_ are the shape and scale parameters of the Gamma distribution. For NND (*SK2-SK2*), NND (*RyR2-RyR2*), and NND (*Ca*_*v*_*1.2-Ca*_*v*_*1.2*), a single Gamma distribution gave the best fit. For NND (*SK2-RyR2*) and NND (*SK2-Ca*_*v*_*1.2*), a mixture of two Gamma distributions gave the best result. The use of two Gamma distributions validates the presence of functional coupling between SK2 and RyR2 as well as SK2 and Ca_v_1.2. Additionally, one can test if the channels are randomly distributed by comparing their distribution to an exponential distribution. In the case of the Gamma distribution, this amounts to comparing *α*_*i*_ to 1; the closer *α*_*i*_ is to 1, the more exponential the distribution of NND becomes, and therefore the more randomly distributed the channels are. Randomly distributed channels will have no functional or spatial relationship between them.

### Patch-clamp recordings

Whole-cell SK currents were recorded using conventional voltage-clamp technique. For recording of the apamin-sensitive currents under a fixed 500 nM free cytosol Ca^2+^ concentration, the control extracellular solution contained (in mM): *N*-methylglucamine (NMG) 140, KCl 4, MgCl_2_ 1, glucose 5, and HEPES 10, pH 7.4 with HCl. The internal solution contained (in mM): potassium gluconate 144, MgCl_2_ 1.15, EGTA 5, HEPES 10, and CaCl_2_ yielding a free cytosol Ca^2+^ concentration of 500 nM using the software by C. Patton of Stanford University (http://maxchelator.stanford.edu/). The pH was adjusted to 7.25 using KOH. To isolate apamin-sensitive SK currents, extracellular solutions containing different concentrations of apamin were applied during the recordings, and the difference currents between the control and the apamin-containing solution were calculated to be the apamin-sensitive currents. The current was elicited from a holding potential of −55 mV using a family of voltage steps from −120 to + 60 mV with a 10-mV increment and 500-ms in duration.

For recording the SK currents activated by Ca^2+^ influx and Ca^2+^ released from SR, the extracellular solution contained (in mM): NMG 130, KCl 1, glucose 10, 4-amiopyridine 5, CaCl_2_ 2, niflumic acid 0.05, E4031 0.001, chromanol 0.01, BaCl_2_ 0.05, and HEPES 10, pH 7.4 with glutamic acid. The pipette solution consisted of (in mM): KCl 45, NMG 120, 10 HEPES, EGTA (or BAPTA) 0.05, pH 7.25 adjusted by glutamic acid. The voltage protocol was designed to isolate the SK currents as shown in Fig. 2.

All experiments were performed using 3 M KCl agar bridges. Cell capacitance was calculated as the ratio of total charge (the integrated area under the current transient) to the magnitude of the pulse (20 mV). Currents were normalized to cell capacitance to obtain the current density. The series resistance was compensated electronically. In all experiments, a series resistance compensation of ≥90% was obtained. Currents were recorded using Axopatch 200 A amplifier (Molecular Devices, LLC., Sunnyvale, CA, USA), filtered at 1 kHz using a 4-pole Bessel filter and digitized at a sampling frequency of 5 kHz. Data acquisition and analysis were carried out using pClamp 10 software (Molecular Devices) and Origin 6.1 software (OriginLab, Northampton, MA, USA).

### Statistical analyses

Where appropriate, pooled data are presented as mean ± S.E.M. Significant differences between the control and the experimental groups were tested using student’s *t*-test. P values of ≤0.05 were considered significant unless otherwise indicated.

## Electronic supplementary material

Supplementary Information
