# Supplementary material for: Coupling of SK channels, L-type Ca2+ channels, and ryanodine receptors in cardiomyocytes
Source: Sci Rep. 2018 Mar 16;8:4670. doi: 10.1038/s41598-018-22843-3 (PMC5856806; doi:10.1038/s41598-018-22843-3)
Supplement: Supplementary file 1 — Supplementary Information [file 41598_2018_22843_MOESM1_ESM.docx]

**Supplemental Information**

**Coupling of SK channels, L-type Ca^2+^ channels, and ryanodine receptors in cardiomyocytes**

**Authors:** Xiao-Dong Zhang^1,2^*, Zana A. Coulibaly^3^, Wei Chun Chen^1^, Hannah A. Ledford^1^, Jeong-Han Lee^4^, Padmini Sirish^1^, Gu Dai^1^, Zhong Jian^3^, Frank Chuang^5^, Ingrid Brust-Mascher^6^, Ebenezer N. Yamoah^4^, Ye Chen-Izu^3^, Leighton T. Izu^3^, Nipavan Chiamvimonvat^1,2^*

**Affiliations:**

^1^Division of Cardiovascular Medicine, Department of Internal Medicine, School of Medicine, University of California, Davis, Davis, CA 95616

^2^Department of Veterans Affairs, Northern California Health Care System

Mather, CA 95655

^3^Department of Pharmacology, School of Medicine, University of California, Davis,

Davis, CA 95616

^4^Department of Physiology and Cell Biology, University of Nevada, Reno, Reno, NV 95616

^5^Department of Biochemistry & Molecular Medicine, University of California, Davis

Sacramento, CA 95817

^6^Health Sciences District Advanced Imaging Facility, University of California, Davis, Davis, CA 95616

*To whom correspondence should be addressed: Division of Cardiovascular Medicine, Department of Internal Medicine, University of California, Davis, One Shields Avenue, GBSF 6315, Davis, CA 95616 & Department of Veterans Affairs, Northern California Health Care System, 10535 Hospital Way Mather, CA 95655. Email: nchiamvimonvat@ucdavis.edu and [xdzhang@ucdavis.edu](mailto:xdzhang@ucdavis.edu)


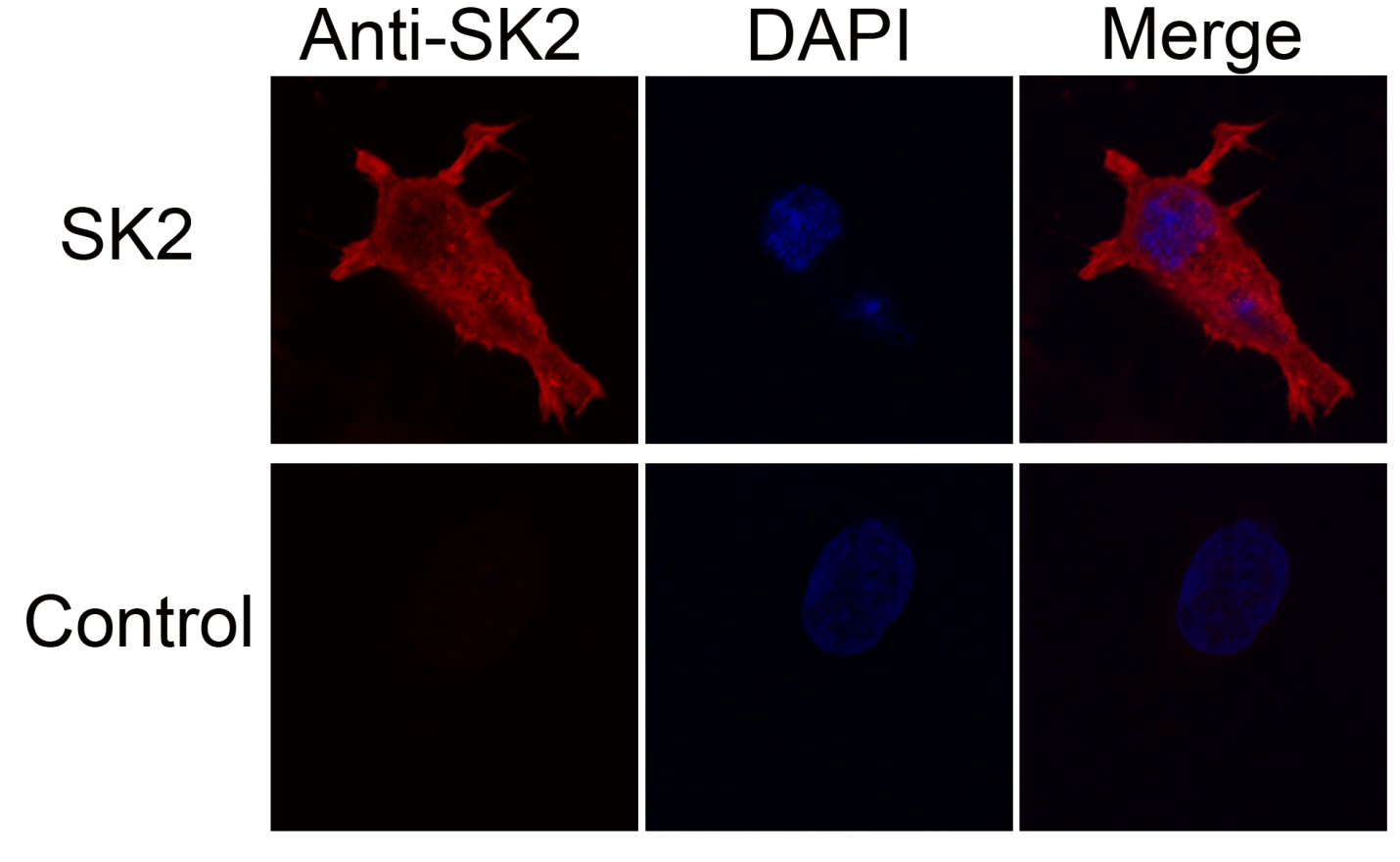


**Supplemental Figure 1**. Specificity testing of anti-SK2 antibody (Abcam 111939, Cambridge, MA, USA) in Human Embryonic Kidney (HEK) 293 cells overexpressing human cardiac SK2 channels. The upper panels labeled SK2 show immunofluorescence confocal laser scanning microscopic images of HEK 293 cells overexpressing human cardiac SK2 channels. The lower panels labeled Control show the results of non-transfected HEK 293 cells. DAPI (4',6-diamidino-2-phenylindole) was used to label nuclei.
